# Applying Machine Learning to Identify Anti-Vaccination Tweets during the COVID-19 Pandemic

**DOI:** 10.3390/ijerph18084069

**Published:** 2021-04-12

**Authors:** Quyen G. To, Kien G. To, Van-Anh N. Huynh, Nhung T. Q. Nguyen, Diep T. N. Ngo, Stephanie J. Alley, Anh N. Q. Tran, Anh N. P. Tran, Ngan T. T. Pham, Thanh X. Bui, Corneel Vandelanotte

**Affiliations:** 1 Physical Activity Research Group, Appleton Institute, Central Queensland University, Rockhampton, QLD 4701, Australia; s.alley@cqu.edu.au (S.J.A.); c.vandelanotte@cqu.edu.au (C.V.); 2 Public Health Faculty, University of Medicine and Pharmacy at Ho Chi Minh City, Ho Chi Minh City 700000, Vietnam; togiakien@ump.edu.vn (K.G.T.); huynhngocvananh@gmail.com (V.-A.N.H.); ntngocdiep1011@gmail.com (D.T.N.N.); quynhanhmo2105@gmail.com (A.N.Q.T.); phuonganhtran3138@gmail.com (A.N.P.T.); thanhnganphamump98@gmail.com (N.T.T.P.); thanhbuivt1998@gmail.com (T.X.B.); 3 Trung Vuong Hospital, Ho Chi Minh City 700000, Vietnam; ntqnhungyhdp14@ump.edu.vn

**Keywords:** deep learning, neural network, LSTM, BERT, transformer, stance analysis, vaccine

## Abstract

Anti-vaccination attitudes have been an issue since the development of the first vaccines. The increasing use of social media as a source of health information may contribute to vaccine hesitancy due to anti-vaccination content widely available on social media, including Twitter. Being able to identify anti-vaccination tweets could provide useful information for formulating strategies to reduce anti-vaccination sentiments among different groups. This study aims to evaluate the performance of different natural language processing models to identify anti-vaccination tweets that were published during the COVID-19 pandemic. We compared the performance of the bidirectional encoder representations from transformers (BERT) and the bidirectional long short-term memory networks with pre-trained GLoVe embeddings (Bi-LSTM) with classic machine learning methods including support vector machine (SVM) and naïve Bayes (NB). The results show that performance on the test set of the BERT model was: accuracy = 91.6%, precision = 93.4%, recall = 97.6%, F1 score = 95.5%, and AUC = 84.7%. Bi-LSTM model performance showed: accuracy = 89.8%, precision = 44.0%, recall = 47.2%, F1 score = 45.5%, and AUC = 85.8%. SVM with linear kernel performed at: accuracy = 92.3%, Precision = 19.5%, Recall = 78.6%, F1 score = 31.2%, and AUC = 85.6%. Complement NB demonstrated: accuracy = 88.8%, precision = 23.0%, recall = 32.8%, F1 score = 27.1%, and AUC = 62.7%. In conclusion, the BERT models outperformed the Bi-LSTM, SVM, and NB models in this task. Moreover, the BERT model achieved excellent performance and can be used to identify anti-vaccination tweets in future studies.

## 1. Introduction

Vaccination is one of the most important public health achievements that save millions of lives annually and helps reduce the incidence of many infectious diseases, including eradicating smallpox [1]. However, anti-vaccination attitudes still exist in the population. A study by the American Academy of Pediatrics showed that 74% of pediatricians encountered a parent who declined or postponed at least one vaccine in a 12-month period [2]. In addition, the prevalence of non-medical vaccination exemption has increased in the last two decades, especially in states with less strict exemption criteria in the U.S. [3]. Vaccine hesitancy was also named as one of the top ten threats to global health by the World Health Organisation in 2019 [4]. During the COVID pandemic, resulting in more than 120 million infections, 2.66 million deaths (as of 17 March 2021), and the development of safe and effective vaccines, it is expected that most people would be willing to vaccinate. However, a study in New York showed that only 59% reported that they would get a vaccine and 53% would give it to their children [5]. Other surveys in Australia showed a higher willingness to vaccinate, about 85% [6] and 75% [7].

The increasing use of social media as a source of health information may contribute to vaccine hesitancy due to anti-vaccination content being widely available on social media [8]. A report found that about 31 million people were following Facebook accounts of ‘anti-vaxxers’ in 2019, and about 17 million people were subscribing to similar accounts on YouTube [9]. Since then, the number of people following anti-vaxxer accounts on social media has increased by at least 7.8 million people [9]. The report also pointed out that those who received information on the COVID pandemic from social media were more likely to be more hesitant about the vaccine [9]. Another study found that uptake of influenza vaccine was inversely associated with the use of Twitter and Facebook for health information [10]. 

Research that can make use of the huge amount of rich data generated from social media, such as Twitter, will be able to provide useful information for formulating strategies that could help reduce anti-vaccination sentiments among different groups. One of the first tasks in this context is to develop a text classification method that can identify anti-vaccination tweets on Twitter. However, given the text-based format and the large amount of data, it is quite a challenging task to handle. An effective approach that was adopted in several Twitter studies on anti-vaccination was to use machine learning techniques. However, most of these studies used traditional machine learning techniques such as support vector machine (SVM), naïve Bayes (NB), and decision tree [11,12,13,14,15,16]. A few other studies did not describe what machine learning techniques they used [17,18] whereas one study used hashtag scores instead of a machine learning technique [19]. Although these methods may generate comparable results in some machine learning tasks compared to deep learning (or deep neural network) [20,21]. Deep learning has been shown to produce state-of-the-art results in many natural language processing tasks [22]. However, only two studies applied deep learning to identify tweets against HPV vaccines [23,24].

Therefore, this study aims to evaluate the performance of different natural language processing models to identify anti-vaccination tweets that were published during the COVID-19 pandemic with the main focus on the bidirectional long short-term memory networks with GLoVe embeddings [25] (Bi-LSTM) and bidirectional encoder representations from transformers (BERT). We also compared the performance of these models with those of classic machine learning methods including SVM and NB. The finding from this study provides useful information to determine an appropriate model for use to identify anti-vaccination tweets in future studies.

## 2. Related Work

Zhou et al. (2015) [15] used a random sample of 884 tweets to develop a supervised classifier that could identify anti-vaccine tweets. Particularly, the SVM method with a radial basis function kernel was used. Forward selection and backward elimination were used to select features that were most likely to discriminate between the two classes. Using only the content of the tweet, the top performer achieved an accuracy of 89.8%.

Mitra et al. (2016) [14] also developed a vaccination stance classifier by training an SVM with 8000 tweets. However, they only used tweets with the same ratings by all three raters as well as only retained tweets with a predicted probability greater than 90%. The accuracy of this classifier was 84.7%.

Another study using SVM was conducted by Shapiro et al. (2017) [13]. However, the classification was implemented in two stages. First, they used 1000 manually labeled tweets to develop a binary classifier that could identify tweets expressing concerns or no concerns about vaccines. This classifier achieved an F1-score of 93% for concern and 81% for non-concern. Then they used another 1000 manually labeled tweets to build another classifier that could identify tweets with specific types of concerns. The performance of this classifier was widely different with F1 scores ranging from 0.22 to 0.92 for each type of concern.

Kunneman et al. (2020) [16] used multinomial naïve Bayes and SVM with a linear kernel to develop a vaccine stance classifier. The classifier was trained on 8259 labeled tweets. The results suggested that SVM as a binary classifier outperformed NB for the task with the highest F1-score of 34% for SVM and 27% for NB. The highest AUC was 63% for SVM and 58% for NB.

Du et al. (2017) [26] also found that SVM outperformed NB and random forest on the ability to identify negative tweets against HPV vaccines. The SVM models used a radial basis function kernel and were trained with 6000 labeled tweets. Compared with the standard SVM model (a micro-averaging F1 score of 67.32%), the hierarchical classification SVM model achieved a micro-averaging F1 score of 72.08%.

For the purpose of identifying anti-vaccination tweets, we found two studies that developed deep learning models with the use of transfer learning. Du et al. (2020) [23] compared the performance of extremely randomized trees (a classic machine learning method) with deep-learning-based methods including Attention-based RNN, Attention-based ELMo, and BERT. The models were developed using 6000 labeled HPV-related tweets. The results showed that top performers were deep-learning-based models with the mean F1 score between 70% and 81%. The other study was conducted by Zhang et al. (2020) [24]. This study used deep learning models with three transfer learning approaches. The first was to use static embeddings (Word2Vec, GloVe, and FastText) [27] and embeddings from language models (ELMo) [28] processed by the bidirectional gated recurrent unit with attention. The other two were to fine-tune generative pre-training (GPT) and BERT models. 6000 tweets relating to HPV were used for the experiments. The results showed that the BERT model was the top performer with a micro-average F1 score of 76.9%.

## 3. Methods

### 3.1. Data Source

Twitter is a social networking platform where users post messages and respond to messages from other users. These messages are known as tweets. A tweet has an original length of 140 characters but since November 2017, the length was doubled to 280 characters [29]. A Twitter dataset collected by Banda et al. 2020 was used [30]. Details of the dataset (version 24) were published elsewhere [30]. In brief, tweets were collected between 1 January and 23 August 2020 using a Twitter Stream API which allows public access to a one percent sample of the daily stream of Twitter. Although the dataset includes 635,059,608 tweets and retweets in the full version, the clean version (no retweets) with 150,657,465 tweets was used. After removing tweets not in English, 75,797,822 tweets were hydrated using the Tweepy library in Python 3 (https://www.tweepy.org, accessed on 10 April 2021). A total of 1,651,687 tweets containing “vaccin”, “vaxx”, or “inocul” were extracted.

### 3.2. Data Processing and Labeling

Texts were changed to lowercase. Twitter handles, URLs, hyphens, hashtags (with attached words), numbers, and special characters were removed. A list of English stop words (e.g., is, that, has, a, do, etc.) from the NLTK library (https://www.nltk.org, accessed on 10 April 2021) were used to remove stop words from the tweets (negations including “not” and “no” were not removed given the purpose was to identify anti-vaccination tweets). Lemmatization, a process of generating the canonical form of a word, was implemented for words in all tweets. Tweets with no content after being processed were removed. A total of 1,474,276 remained.

A systematic random sampling method was used to select 20,854 tweets from 1,474,276 tweets for labeling. This sampling method ensures that tweets across the different times during the pandemic were selected. Tweets were labeled as either “anti-vaccination” or “other” (i.e., neutral, news, or ambiguous) as the model was aimed to use for stance analysis. In stance analysis, a tweet is determined to be in favor or against a target [31]. This is different from sentiment analysis in which a tweet is classified as positive or negative. A negative tweet may not mean anti-vaccine while a positive tweet may not mean pro-vaccine. Ten researchers worked in pairs to label the tweets. Differences in labeling were checked and decided by a third researcher. The average agreement between the two raters was 91.04% ranging between 85.90% and 94.48% (Supplementary file). The percentage of anti-vaccine tweets was 9.1%. The data were then split into three parts: training set (70%), development set (15%), and test set (15%). The training and development sets were used to build the model, the performance of which was evaluated on the test set.

### 3.3. Bidirectional Long Short-Term Memory Networks (Bi-LSTM)

Recurrent neural networks (RNN) have been used in many natural language processing tasks due to their ability to handle sequential data with various lengths. However, standard RNNs have limitations. First, as the inputs are processed in order, the outputs are mostly based on only previous context (i.e., words) [32]. The second issue is referred to as difficulty in learning long-term dependencies when the sentences are too long [32,33]. For the first problem, a solution is to use bidirectional RNN [32,34]. Bidirectional RNNs combine two unidirectional RNNs that process data in two opposite directions. As such, at every time step, the bidirectional RNN has all information before and after it [34]. For the second problem, LSTM units can be used. An LSTM unit is comprised of a cell that can remember information over time intervals, and a set of gates (i.e., input, forget, and output gates) that are used to control which information flows into and out of the cell [32,35]. Additionally, word embeddings from pre-trained models were used to increase performance. Specifically, we used the GloVe model, pre-trained with 2 billion tweets, 27 billion tokens, and 200 dimensions [25].

The RNN with one bidirectional LSTM layer was used as increasing the network size did not improve the performance. We used a dropout rate of 0.1, Adam with weight decay (AdamW) optimizer, and binary cross-entropy loss function. We also experimented with a learning rate = (0.00003, 0.0001, 0.001), the number of units of the bidirectional LSTM layer = (256, 128, 64), and the number of epochs = (10, 20, 30, 40, 50, 60, 70, 80). Class weights were also calculated and used in the training.

### 3.4. Bidirectional Encoder Representations from Transformers (BERT)

Although static word embedding methods such as GloVe and word2vec have obtained great achievement in many natural language processing tasks, it does not take into account the order of words in the sentence. Also, the same word may have different meanings depending on the context of the sentence. This problem is addressed with dynamic embedding methods such as BERT [36] that produce vector representations for words conditional on the sentence context. BERT has been shown to achieve new state-of-the-art results on natural language processing tasks [36]. In this study, we used the BERT pre-trained uncased model with 12 hidden layers (transformer blocks), a hidden size of 768, and 12 attention heads (https://tfhub.dev/tensorflow/bert_en_uncased_L-12_H-768_A-12/3, accessed on 10 April 2021). We also experimented with different learning rate = (0.0003, 0.0001) and number of epochs = (1, 2, 3, 4, 5).

### 3.5. Support Vector Machine (SVM) and Naïve Bayes (NB) Classifier

SVM [37] and NB [38] are traditional machine learning methods that have been used in text classification tasks [13,14,15]. Some studies showed that the performance of SVM and NB is comparable to neural networks [20,21] while the opposite results were found in the other studies [39,40]. In this study, we used the term frequency-inverse document frequency method to vectorize the text data. In addition, we experimented with four SVM kernels = [linear, poly, radial basis function, and sigmoid] but used default values (as reported in c-support vector classification, the Scikit-learn package) for other parameters. For NB, we used the complement NB and multinomial NB.

### 3.6. Metrics for Evaluating Performance

We reported the following metrics for evaluating the performance of all machine learning models. Accuracy is the proportion of tweets correctly predicted by the model over all of the tweets. Precision (also named positive predictive value) is the proportion of anti-vaccination tweets that are correctly predicted by the model over all anti-vaccination predictions. Recall (also named sensitivity) is the proportion of anti-vaccination tweets that are correctly predicted by the model over all anti-vaccination tweets. As the data are imbalanced (i.e., the percentage of anti-vaccination tweets is small), accuracy may not be a good metric. Therefore, we used the F1 score as the primary metric. We also reported the area under the receiver operating characteristic curve (AUC) which is drawn based on true positive and false-positive rates.

(1) $$F1\;score=2\;\times \;\frac{Precision\;\times \;Recall}{Precision\;+\;Recall}$$

(2) $$Accuracy=\;\frac{True\;positive+true\;negative\;}{Total\;number\;of\;predictions}$$

(3) $$Precision=\;\frac{True\;positive\;}{True\;positive+false\;positive}$$

(4) $$Recall=\;\frac{True\;positive}{True\;positive+false\;negative}$$

## 4. Results

Table 1 shows the performance of the Bi-LSTM models on the development set. We only reported results for Bi-LSTM models with 128 units as these outperformed those with 64 and 256 units. In general, the performance of these 128-unit models was not very different across learning rates and epochs. The top performer was the Bi-LSTM-128 model that used a learning rate of 0.0001 and was trained for 60 epochs. For this model, the F1 score was 51.7%. AUC was also quite high (87.9%).

Table 2 shows the performance of the BERT models on the development set. In general, all BERT models performed very well. F1 scores for all models were above 95%. Although AUC was also high, the models seem to overfit after three epochs. The top performer based on the F1 score was the model which was trained with a learning rate of 0.0001 and for 3 epochs.

Table 3 shows the performance of the SVM and NB models on the development set. The SVM model with linear kernel outperformed the other SVM models with an F1 score of 32.2% and AUC of 83.9%. The complement NB model, which achieved an F1 score of 30.5% and AUC of 65.2%, outperformed the multinomial NB model. Although F1 scores were similar between the SVM model with linear kernel and the complement NB (32.2% vs. 30.5%, respectively), the SVM model with linear kernel achieved much higher AUC compared to the complement NB (83.9% vs. 65.2%, respectively).

Table 4 shows the performance of the top Bi-LSTM, BERT, SVM, and NB models that were evaluated on the test set. The BERT model outperformed the other models with an F1 score of 95.5% which is more than two times higher than the Bi-LSTM model (45.5%) and three times higher than the SVM with the linear kernel (31.2%) and the complement NB (27.1%) models. However, the performance of AUC for the BERT model was lower when evaluating with the test set (84.7%) compared to the development set (90.8%). AUC for the complement NB model was also low at 62.7%.

## 5. Discussion

This study aimed to evaluate the performance of machine learning models on identifying anti-vaccination tweets that were obtained during the COVID-19 pandemic. The findings showed that BERT models outperformed the Bi-LSTM, SVM, and NB models across all performance metrics (i.e., accuracy, precision, recall, F1 score, and AUC). The next top performer was the Bi-LSTM deep learning models. Classic machine learning models including SVM and NB models did not perform as well on this task of identifying the anti-vaccination tweets compared to the BERT and Bi-LSTM models.

The BERT models did very well on this text classification task with four of five metrics being above 90% and an AUC of 84.7%. This is higher than the performance of systems using the classic SVM method (accuracy less than 90%) [14,15,18]. Our finding is consistent with other studies that deep learning-based models outperformed classic machine learning methods on this task [23,24]. Moreover, the finding that BERT models outperformed other deep learning models is consistent with that by Zhang et al. (2020) [24]. The BERT model also achieved an F1 score higher than the deep learning models by Du et al. (2020) (mean F1 scores from 70% to 81%) [23] and Zhang et al. (2020) (F1 score 76.9%) [24]. These results show that the BERT models were extremely good at identifying anti-vaccination tweets even in the case that the data are imbalanced (i.e., anti-vaccination tweets were a small percentage of all vaccination tweets). With a basic BERT model, we have been able to achieve an F1 score higher than F1 scores achieved by a more complex static word embedding system, which was the top performer (average F1 score of 67.8%) among the 19 submissions to a supervised stance analysis task [41]. We suggest that the BERT model should be considered as a method of choice for stance analysis on large Twitter datasets. This finding is not surprising given that the BERT model has been shown to outperform other state-of-the art natural language processing systems and even human performance on eleven natural language processing tasks [36].

In this study, the average agreement rate between coders (91.04%) was comparable to that in other studies which were 85.1% by Du et al., 2020 [23], 95% by Zhou et al., 2015 [15], and 100% by Tomeny et al., 2017 [18]. However, the number of tweets used in this study (20,854 tweets) was larger than those used in other studies such as 884 tweets by Zhou et al., 2015 [15], 2000 tweets by Tomeny et al., 2017 [18], 6000 tweets by Du et al., 2020 [23], and 8000 tweets by Mitra et al., 2016 [14] which is a strength of this study.

This study has some limitations. As public access to tweets is limited due to rules imposed by Twitter, the tweets used in this study accounted for only one percent of daily tweets and therefore, may not be representative for all of the tweets. In addition, due to lack of time and resources needed for training, model fine-tuning was limited to a few learning rates and the number of epochs, other parameters were not tuned. The performance of these models might have been improved further if the tuning had been conducted more widely. However, we consider that the performance of BERT models in this study was excellent and good enough for use to identify anti-vaccination tweets in future studies.

## 6. Conclusions

The BERT models outperformed the Bi-LSTM, SVM, and NB models on this task. Moreover, the BERT model achieved excellent performance and can be used to identify anti-vaccination tweets in future studies.

## Figures and Tables

**Table 1:** Performance of the Bi-LSTM-128 models on the development set.

Learning Rate	Epoch	Accuracy	Precision	Recall	F1 Score	AUC
0.00003	10	77.2%	26.2%	74.9%	38.9%	84.2%
	20	78.7%	28.1%	76.9%	41.1%	85.1%
	30	79.8%	28.7%	73.3%	41.2%	86.3%
	40	82.3%	31.2%	68.6%	42.9%	87.1%
	50	82.3%	31.3%	69.3%	43.2%	87.6%
	60	82.7%	31.8%	68.6%	43.4%	87.7%
	70	81.3%	30.1%	70.6%	42.2%	87.6%
	80	82.5%	31.9%	71.3%	44.0%	88.0%
0.0001	10	80.7%	29.9%	73.9%	42.6%	87.2%
	20	80.5%	29.9%	75.6%	42.8%	88.3%
	30	80.8%	30.1%	74.9%	43.0%	88.0%
	40	85.8%	37.0%	66.3%	47.5%	88.2%
	50	89.7%	47.2%	53.1%	50.0%	86.2%
	**60**	**88.4%**	**43.2%**	**64.4%**	**51.7%**	**87.9%**
	70	86.1%	37.4%	64.0%	47.2%	87.3%
	80	88.8%	43.9%	54.1%	48.4%	85.0%
0.001	10	84.5%	35.2%	71.3%	47.1%	88.1%
	20	84.7%	35.1%	68.0%	46.3%	86.9%
	30	90.0%	47.9%	41.6%	44.5%	83.8%
	40	89.1%	44.6%	52.1%	48.1%	80.4%
	50	90.7%	53.3%	34.3%	41.8%	74.5%
	60	89.6%	46.2%	42.6%	44.3%	78.3%
	70	88.6%	42.1%	47.2%	44.5%	79.4%
	80	88.7%	42.5%	46.5%	44.4%	77.5%

**Table 2:** Performance of the BERT models on the development set.

Learning Rate	Epoch	Accuracy	Precision	Recall	F1 Score	AUC
0.00003	1	91.7%	92.5%	98.8%	95.5%	90.7%
	2	91.8%	94.3%	96.8%	95.5%	91.5%
	3	92.2%	94.6%	96.8%	95.7%	86.5%
	4	92.1%	94.5%	96.9%	95.7%	83.7%
	5	91.7%	94.5%	96.4%	95.4%	79.8%
0.0001	1	92.1%	93.5%	98.0%	95.7%	91.0%
	2	92.0%	94.1%	97.2%	95.6%	91.4%
	**3**	**92.5%**	**94.5%**	**97.3%**	**95.9%**	**90.8%**
	4	92.1%	94.5%	96.9%	95.7%	84.6%
	5	92.0%	94.4%	96.9%	95.6%	82.1%

**Table 3:** Performance of the SVM and NB models on the development set.

	Accuracy	Precision	Recall	F1 Score	AUC
SVM-linear	91.7%	20.5%	75.6%	32.2%	83.9%
SVM-poly	90.8%	9.2%	66.7%	16.2%	78.9%
SVM-rbf	91.1%	12.5%	74.5%	21.5%	83.0%
SVM-sigmoid	91.5%	17.5%	75.7%	28.4%	83.8%
Complement NB	88.8%	25.4%	38.1%	30.5%	65.2%
Multinomial NB	90.6%	5.6%	68.0%	10.4%	79.4%

**Table 4:** Performance among Bi-LSTM, BERT, SVM, and NB on the test set.

	Accuracy	Precision	Recall	F1 Score	AUC
Bi-LSTM-128, learning rate = 0.0001, epoch = 50	89.8%	44.0%	47.2%	45.5%	85.8%
BERT, learning rate = 0.0001, epoch = 3	91.6%	93.4%	97.6%	95.5%	84.7%
SVM-linear	92.3%	19.5%	78.6%	31.2%	85.6%
Complement NB	88.8%	23.0%	32.8%	27.1%	62.7%

## Supplementary Materials

The following are available online at https://www.mdpi.com/article/10.3390/ijerph18084069/s1, Confusion Matrix Tables, and Examples of Prediction.

## Abbreviations

AUC	Area under the receiver operating characteristic curve
BERT	Bidirectional encoder representations from transformers
Bi-LSTM	Bidirectional long short-term memory networks
ELMo	Embeddings from language models
GPT	Generative pre-training
NB	Naïve Bayes
RNN	Recurrent neural networks
SVM	Support vector machine
