# Supplementary material for: Applying Machine Learning to Identify Anti-Vaccination Tweets during the COVID-19 Pandemic
Source: Int J Environ Res Public Health. 2021 Apr 12;18(8):4069. doi: 10.3390/ijerph18084069 (PMC8069687; doi:10.3390/ijerph18084069)
Supplement: Supplementary file 1 [file ijerph-18-04069-s001.pdf]

Supplementary file

Confusion matrix tables:.

		Rater 1		
		Not anti-vaccine	Anti-vaccine	Total
Rater 2	Not anti-vaccine	273	0	273
	Anti-vaccine	18	8	26
	Total	291	8	299

		Rater 1		
		Not anti-vaccine	Anti-vaccine	Total
Rater 2	Not anti-vaccine	781	19	800
	Anti-vaccine	122	78	200
	Total	903	97	1000

		Rater 1		
		Not anti-vaccine	Anti-vaccine	Total
Rater 2	Not anti-vaccine	821	24	845
	Anti-vaccine	105	50	155
	Total	926	74	1000

		Rater 1		
		Not anti-vaccine	Anti-vaccine	Total
Rater 2	Not anti-vaccine	843	47	890
	Anti-vaccine	40	70	110
	Total	883	117	1000

		Rater 1		
		Not anti-vaccine	Anti-vaccine	Total
Rater 2	Not anti-vaccine	826	66	892
	Anti-vaccine	37	68	105
	Total	863	134	997

		Rater 1		
		Not anti-vaccine	Anti-vaccine	Total
Rater 2	Not anti-vaccine	807	59	866
	Anti-vaccine	55	76	131
	Total	862	135	997

		Rater 1		
		Not anti-vaccine	Anti-vaccine	Total
Rater 2	Not anti-vaccine	1302	64	1366
	Anti-vaccine	106	151	257
	Total	1408	215	1623

		Rater 1		
		Not anti-vaccine	Anti-vaccine	Total
Rater 2	Not anti-vaccine	854	32	886
	Anti-vaccine	66	45	111
	Total	920	77	997

		Rater 1		
		Not anti-vaccine	Anti-vaccine	Total
Rater 2	Not anti-vaccine	913	17	930
	Anti-vaccine	46	21	67
	Total	959	38	997

		Rater 1		
		Not anti-vaccine	Anti-vaccine	Total
Rater 2	Not anti-vaccine	825	85	910
	Anti-vaccine	20	67	87
	Total	845	152	997

		Rater 1		
		Not anti-vaccine	Anti-vaccine	Total
Rater 2	Not anti-vaccine	887	18	905
	Anti-vaccine	53	39	92
	Total	940	57	997

		Rater 1		
		Not anti-vaccine	Anti-vaccine	Total
Rater 2	Not anti-vaccine	918	40	958
	Anti-vaccine	15	24	39
	Total	933	64	997

		Rater 1		
		Not anti-vaccine	Anti-vaccine	Total
Rater 2	Not anti-vaccine	829	74	903
	Anti-vaccine	29	65	94
	Total	858	139	997

		Rater 1		
		Not anti-vaccine	Anti-vaccine	Total
Rater 2	Not anti-vaccine	865	37	902
	Anti-vaccine	61	34	95
	Total	926	71	997

		Rater 1		
		Not anti-vaccine	Anti-vaccine	Total
Rater 2	Not anti-vaccine	844	30	874

	Anti-vaccine	63	60	123
	Total	907	90	997

		Rater 1		
		Not anti-vaccine	Anti-vaccine	Total
Rater 2	Not anti-vaccine	861	25	886
	Anti-vaccine	64	47	111
	Total	925	72	997

		Rater 1		
		Not anti-vaccine	Anti-vaccine	Total
Rater 2	Not anti-vaccine	869	39	908
	Anti-vaccine	32	57	89
	Total	901	96	997

		Rater 1		
		Not anti-vaccine	Anti-vaccine	Total
Rater 2	Not anti-vaccine	879	39	918
	Anti-vaccine	40	39	79
	Total	919	78	997

		Rater 1		
		Not anti-vaccine	Anti-vaccine	Total
Rater 2	Not anti-vaccine	882	33	915
	Anti-vaccine	38	44	82
	Total	920	77	997

		Rater 1		
		Not anti-vaccine	Anti-vaccine	Total
Rater 2	Not anti-vaccine	897	13	910
	Anti-vaccine	51	36	87
	Total	948	49	997

		Rater 1		
		Not anti-vaccine	Anti-vaccine	Total
Rater 2	Not anti-vaccine	870	58	928
	Anti-vaccine	20	49	69
	Total	890	107	997

Examples of prediction

Anti-vaccine	*#coronavirus Watch the elites. If they don't succumb at rates similar to the rest of the population, suspect a vaccine in use. Watch the Davos crowd, to see if this is a Globalist depopulation stunt.*
	*The #MSM will use this so called #coronavirus in order to promote toxic autism causing #vaccines f*** globalists #AmericaFirst*
	@99freemind It would make complete sense that the whole coronavirus story is a racket with the goal of scaring a large part of the world population to willingly get "experimental" vaccine shots with serious side-effects that are actually the hidden main-effect.
Not anti-vaccine	*Primary care has always been free regardless of immigration status, but most people without status aren't registered with a GP.*<br><br>*@DHSCgovuk must go further so that everyone can access the Covid-19 vaccine, regardless of immigration status. #VaccinesForAll*<br>*https://t.co/4x9TVEIj4s*
	This coronavirus isn't new. Seems it was to be used in vaccines etc from earlier on in the decade. https://t.co/boJMyGhGqD
	Lebanon races to vaccinate population as COVID-19 deaths rise https://t.co/6uGolG7XMn #politics #feedly
